# Editorial: Into the heart of systemic autoimmune diseases

**DOI:** 10.3389/fmed.2024.1392487

**Published:** 2024-03-18

**Authors:** Micaela Fredi, Silvia Piantoni, Antonio Brucato, Franco Franceschini

**Affiliations:** ^1^Rheumatology and Clinical Immunology Unit, Department of Clinical and Experimental Sciences, Azienda Socio-Sanitaria Territoriale (ASST) Spedali Civili and University of Brescia, Brescia, Italy; ^2^Department of Biomedical and Clinical Sciences, University of Milano, Fatebenefratelli Hospital, Milano, Italy

**Keywords:** autoimmunity, cardiovascular risk, inflammation, biomarkers, cardiovascular assessment

Autoimmune rheumatic diseases are further burdened by cardiovascular complications (1). The amount of risk for each patient, who deserve personalized monitoring for cardiovascular comorbidities, is related to two main factors: the effects of the underlying inflammatory mechanisms of the disease, which have a direct action on the cardiovascular system (2) and the lengthening of life expectancy due to new therapeutic interventions (3). In fact, the traditional cardiovascular risk factors, which act in the general population, are also largely found in our patients (3). Currently, the main pillars of cardiovascular risk reduction are the pharmacological and non-pharmacological management of the modifiable risk factors, as well as the tight control of disease activity (4).

The aim of this Research Topic of manuscripts was to give an update about the physio-pathological and clinical aspects of the primary or secondary cardiovascular manifestations in systemic autoimmune diseases.

Due to the lack of specific prediction models, clinicians should use scores validated for the general population to screen for cardiovascular risk in autoimmune diseases (4). This Research Topic has been addressed by Mandel et al. in their review. They proposed new surrogate markers of cardiovascular risk, such as arterial stiffness and the parameters obtained from cardiovascular imaging techniques, or soluble markers that were demonstrated to be disease-related. Moschetti et al. focused their review on endothelial dysfunction, which is considered the first inflammation-induced pathogenic event triggering vascular remodeling, at the basis of microangiopathy. They focused on systemic lupus erythematosus and on systemic sclerosis, in which endothelial dysfunction is the main event of pre-clinical atherosclerosis or a key pathogenetic factor at the basis of the disease itself, respectively. Some experience in the use of some of these techniques for the evaluation of cardiovascular risk in autoimmune diseases was described by our group in one of the papers of the collection. Piantoni et al. described the use of adaptive optics, a new tool for the evaluation of retinal arterioles, which represents a good arterial compartment for the study of microcirculation. They demonstrated the reduction of an index, which is a sign of microvasculature alteration, after 12 months of therapy with abatacept, proposing a possible new scenario in the use of biological disease modifying anti-rheumatic drugs (bDMARDs) in rheumatoid arthritis. Lazzerini et al. proposed a specific interesting topic in their review: the role of autoimmunity in the pathogenesis of cardiac arrhythmias. In particular, they underlined that increasing evidence was being reported on the role of the anti-Ro/SSA antibodies in affecting the ventricular repolarization. This is an important point considering the prevalence of these autoantibodies in patients with autoimmune diseases, but also in the general population.

Systemic vasculitis is a heterogenous group of autoimmune diseases, some of which have the cardiovascular system as one of the primary target organs. Two reviews in this Research Topic were dedicated to summarizing novel therapies used in Takayasu arteritis (TAK) and Eosinophilic granulomatosis with polyangiitis (EGPA). As revised by Uzzo et al., TAK is one of the vasculitis with the most frequent heart involvement. The whole aorta and all the aortic branches can be affected, but also cardiac manifestations can appear. High dosage of steroids remains fundamental, but adverse events and possible relapses require the introduction of alternative treatment. Several new therapeutic approaches with bDMARDs and targeted synthetic DMARDs have showed promising results, with high efficacy and acceptable safety profile, although most of the available data are obtained from cohort studies. These results led to the inclusion of anti-TNF alpha therapies as first line therapies in the most recent ACR guidelines (5).

Similarly, also for EGPA in the last decades several new treatments were available, as revised by Regola et al. Cardiac involvement in EGPA is a rare complication of a rare disease, but remains one of the most serious and main causes of death. Historical treatments include high dosage of steroids combined with several conventional DMARDs. However, as revised by the authors, in 2017 the first biological treatment, the anti-IL5 mepolizumab, was approved for EGPA. For both TAK and EGPA several RCTs are ongoing, targeting molecules/cell types involved in the pathogenesis of the diseases.

In the era of the COVID-19 pandemic infection, a small percentage of children have been reported to have developed a serious condition with multi-system organ disfunction, increased inflammatory biomarkers, that was called Multisystem Inflammatory Syndrome in Children (MIS-C). Panaro et al. underlined in their review how this new condition shares several similarities with Kawasaki disease (KD), one of which is cardiac involvement. Moreover, the authors summarized clinical features, pathogenesis, and available treatments as first-line treatment or for refractory patients. As mentioned, cardiac involvement is one of the most severe clinical features of KD, with an increased risk of coronary artery aneurysm or cardiovascular events. Buda et al. reviewed the available treatment guidelines and summarized the standard second-line treatment and drugs used in non-responsive or high-risk patients.

Finally, Ammirati et al. focused their review on two cardiological manifestations that can occur in the context of autoimmune diseases or as isolated forms: acute myocarditis and recurrent/acute pericarditis. Additionally, the authors provide an overview of the available treatments for both conditions and how evolving technologies may guide the use of these treatments.

In conclusion, we believe that the papers included in this Research Topic are excellent examples of the complex and heterogeneous involvement of the cardiovascular system in the field of systemic autoimmune diseases.

## Author contributions

MF: Writing—original draft. SP: Writing—original draft. AB: Writing—review & editing. FF: Writing—review & editing.
